# Progression in Neuroimaging of Normal Pressure Hydrocephalus

**DOI:** 10.3389/fneur.2021.700269

**Published:** 2021-11-18

**Authors:** Rui Yin, Junxian Wen, Junji Wei

**Affiliations:** Department of Neurosurgery, Peking Union Medical College Hospital, Peking Union Medical College, Chinese Academy of Medical Sciences, Beijing, China

**Keywords:** normal pressure hydrocephalus (NPH), neurodegenerative disease, neuroimaging, radiology, artificial intelligence

## Abstract

Normal-pressure hydrocephalus is a clinical syndrome that mainly targets the elderly population. It features dementia, impaired walking, and the malfunction of sphincters. The rapid identification and large-scale screening of patients with normal-pressure hydrocephalus (NPH) are of great significance as surgical interventions can greatly improve or even reverse the symptoms. This review aims to summarize the traditional parameters used to diagnose NPH and the emerging progression in neuroimaging of the disease, hoping to provide an up-to-date overall perspective and summarize the possible direction of its future development.

## Introduction

Normal-pressure hydrocephalus is a concept first raised by Hakim and Adams in 1965, who defined the disease with two elementary ideas—symptomatic ventriculomegaly and cerebrospinal fluid (CSF) pressure within a normal range (<200 mmH_2_O) (1). The disease features three major clinical manifestations—intellectual impairment, gait disturbance, and urinary dysfunction (2). As a special form of communicating hydrocephalus, normal-pressure hydrocephalus (NPH) can be subdivided into idiopathic and secondary cases. Secondary NPH usually has specific causes including hemorrhage and meningitis. However, the initial symptoms of idiopathic NPH (iNPH) may be quite mild and can be easily confused with signs of normal aging. Most patients only show gait changes in the early phase, but will gradually lose the ability to take care of themselves in daily life, which exerts an unimaginable burden on their families (3). Since iNPH can be treated through surgical procedures (e.g., ventriculoperitoneal or lumboperitoneal shunting), and early intervention is associated with a better chance of a favorable prognosis (4), it is of great significance to precisely identify and diagnose patients with NPH as early as possible. A valid diagnosis of NPH should be established based on clinical presentation, radiological findings, and diagnostic tests. Currently, there are no standard diagnostic criteria for NPH imaging. Besides hydrocephalus, an enlarged ventricle can also be attributed to degenerative neurological diseases like brain atrophy and Alzheimer's disease (AD). There are also illnesses like Parkinson's disease (PD) that shares similar manifestations (e.g., walking and moving difficulties). Therefore, it remains a diagnostic challenge, in many cases, to distinguish iNPH from other neurological disorders (5). Hence in this review, we aimed to focus on literatures on the characteristics and emerging techniques of NPH imaging that can highlight the unique changes of NPH.

## Conventional Morphological Parameters

Generally, both CT and MRI scans can confirm the dilation of ventricles, but if available, an MRI scan is preferred as it can provide more information that can be used to rule out other possible etiology. In addition to visually identifiable ventriculomegaly, some mature structural parameters can be utilized for further analysis.

### Evans' Index (EI)

Evan's index is a widely used indicator for ventriculomegaly, defined as the ratio of the maximum width of the frontal horn of the lateral ventricle over the largest inter diameter of the skull (not the brain) on the same axial level. An EI >0.3 implies the existence of hydrocephalus. Although the index is not precise and informative enough to help with neither differential diagnosis nor prognostication, it is easy to utilize as it can be performed on ordinary CT or MRI scans without instrumental assistance.

### Callosal Angle (CA)

Callosal angle is another radiological feature that can distinguish NPH and atrophy and eliminate non-hydrocephalus patients. The specific measurement of CA depends on the level of the coronal plane selected (perpendicular to the bi-commissural line). Some researchers choose to use the coronal plane at the level of the posterior commissure and discovered that patients with NPH usually have smaller angles (<90°) than normal or atrophy subjects (100–120°), and the measurement may be helpful when selecting suitable patients for shunting surgery as those with smaller CA tend to have a favorable prognosis (6, 7).

### Magnetic Resonance Hydrocephalic Index (MRHI)

In recent research, Andrea et al. described this new biomarker as the largest width of the collateral trigones of the lateral ventricles over the maximum diameter of the inner skull on magnetic resonance (MR) images (T1), aiming to distinguish iNPH with progressive supranuclear palsy (PSP) as these two entities have similar features, both clinically and radiologically. For comparison, they also tested the discriminating power of CA using the same subjects. The authors discovered that MRHI exhibited a surprisingly high accuracy in differentiation (98.5%) compared with CA (positive predictive value of only 70%) (8).

### Disproportionate Enlarged Subarachnoid Space Hydrocephalus (DESH)

As discussed above, EI >0.3 is not unique to iNPH (i.e., low specificity). In addition, clinicians need to first rule out the possibility of cerebral atrophy, which is commonly found in the elder population. According to the Japanese guideline, a feature termed DESH can help with the distinguishment. It is characterized by more crowded sulci near the superior cortex while larger spaces are seen inferiorly, where CSF accumulates. Disproportionate enlarged subarachnoid space hydrocephalus is a promising sign for iNPH diagnosis as well as shunting responsiveness (9–11). Similar findings also exist in Sylvian fissures and focal sulci, that disproportional widening suggests NPH.

### Dilated Third Ventricle

Normal-pressure hydrocephalus, as a special form of communicating hydrocephalus, possesses no obstruction in the flow of CSF. Similar to ordinary individuals, the CSF in iNPH patients flows back and forth through the aqueduct due to cardiac pulse, while a net direction toward the fourth ventricle can be observed. In addition to the lateral ventricles, the third ventricle also dilates such that on the coronal panels, its wall bows outward rather than concaved (as in normal people). This morphological change is also useful in diagnosing iNPH (12).

### Wider Temporal Horns of Lateral Ventricles

The temporal horns, or the inferior horns of the lateral ventricles, project anteriorly and inferiorly into the temporal lobe of the brain. The widening of the horns indicates dilated lateral ventricles and a width of 6 mm or more can be seen in iNPH patients (13).

### Bowling of Corpus Callosum

The corpus callosum is a bundle of C-shaped nerve fibers that sits beneath the cerebral cortex, connecting the left and right cerebral hemispheres. In patients with NPH, the corpus callosum can be found elevated upwards, serving as another structural marker for the diagnosis of NPH (14).

### Net Flow in the Aqueduct

A phase-contrast MRI may be of potential help in predicting shunting responsiveness. As a quantitative method to measure the CSF flow through the aqueduct (in both directions), it can provide precise net volume when paired with cardiac stroke monitoring. A large absolute volume from the third ventricle toward the fourth indicates a better chance of improvement after surgery (12).

While conventional morphological parameters have served as essential biomarkers for the diagnosis and management of iNPH, both in research fields as well as in clinical practices in decades, they still cannot provide valuable information of more depth in the aspect of function or metabolism. There has not been much fundamental breakthrough in morphological clues in recent years. Modern studies focus more on aspects of tissue metabolism, functional changes, elasticity, etc.

## Advanced Imaging Techniques and Modalities on Function and Metabolism

### PET

Positron emission tomography scan investigates the glucose metabolism in any part of the body. Townley et al. did a retrospective study, revealing that patients with iNPH had significant regional hypometabolism in the dorsal striatum, involving the caudate and putamen bilaterally when compared with controls and patients with other comorbidities like AD, dementia with Lewy bodies (DLB)/pervasive developmental disorders (PDD), and behavioral variant of frontotemporal dementia (bvFTD) (15). This conclusion not only sheds light on a more accurate diagnosis of iNPH but also helps distinguish other neurodegenerative diseases apart. Calcagni et al. from Italy used this technique to study the cerebral glucose metabolism in 20 iNPH patients before and after shunt surgery and concluded that clinical improvement depends on the recovery of the CSF dynamic as well as metabolic function restoration (16). Furthermore, Miyazaki et al. further pushed the boundary by concluding that NPH develops when hypometabolism is detected in the basal ganglia, otherwise, preclinical NPH is the diagnosis (17). In addition, when using specific tracers, a PET scan can precisely reflect tau deposition in the brain, distinguishing AD apart (18).

### MR Elastography (MRE)

Fattahi et al. investigated the use of MRE in the diagnosis of NPH (19). As a novel scanning sequence, MRE can reveal brain stiffness using shear waves and post-processing artificial techniques. They observed an increase in brain stiffness in several brain regions of patients with NPH, including the cerebrum, occipital lobe, parietal lobe, and temporal lobe. Unexpectedly, a decrease in the elasticity in the frontal lobe and deep gray/white matter were found in patients with NPH. The underlying mechanism remains unclear. They concluded that MRE is of great potential and needs to be further studied and applied for NPH assessment. The finding is in accordance with that of Perry et al., which concluded altered brain stiffness in iNPH is correlated with clinical symptoms (20). They also suspect that increased temporal stiffness may predict surgical failure and potentially suggest an alternative dementing pathology underlying the iNPH-like symptoms.

### Diffusion Tensor Imaging (DTI)

Diffusion tensor imaging is another non-invasive technique that may be utilized to predict surgical outcomes in patients with iNPH. It is an MRI technique that uses anisotropic diffusion to estimate the white matter organization of the brain. When paired with fiber tractography (FT), a three-dimensional (3D) reconstruction can be achieved to assess neural tracts. Nicole et al. found DTI useful in revealing neural distortion in patients with NPH and helpful in studying the topography and reversibility of white matter injury, which may be associated with the clinical manifestations of NPH (21). Ades-Aron et al. used this technique to gain data from patients with NPH, AD, and healthy controls. Their analysis revealed the differentiating power of DTI, that axial diffusion, axial kurtosis, and the axonal water fraction differ significantly across the groups (22). Unique micro-changes of white matter might explain impairments in patients with NPH, suggesting using DTI results to predict shunt responsiveness in patients with NPH is feasible (23). Hattori et al. concluded that DTI helps distinguish patients with iNPH from those with AD etc. as DTI reveals altered microstructures in the corticospinal tract (CST) (24). A similar idea was proposed by Nakanishi et al. in their study assessing microstructural changes in CST using diffusional kurtosis imaging (25).

### Arterial Spin Labeling

It is inevitable to consider blood supply when dealing with neurologic diseases. It is believed that in patients with iNPH, the cerebral blood flow will decrease (26). This is where new techniques like arterial spin labeling come into use. It labels and tracks the blood perfusing the brain without the need for a contrast agent like gadolinium. According to Virhammar et al., this non-invasive method confirmed the reduced blood supply in the periventricular white matter, basal ganglia, and thalamus in patients with iNPH (27). The conclusion is in joint agreement with the papers produced by Bradley et al. in which the authors found deep white matter ischemia or infarction in iNPH patients based on MRI imaging (28, 29). This idea supposed ischemia as one of the reasons behind the malfunction of CSF reabsorption in NPH patients.

### Glymphatic MRI

The glymphatic system is believed to be crucial to the clearance of the brain waste accumulated in interstitial spaces. Ringstad et al. carried out a controlled study comparing the glymphatic condition in patients with iNPH and non-iNPH neurosurgery patients using MRI. They noted the decreased glymphatic clearance in iNPH patients and hypothesized that reduced glymphatic function is instrumental for dementia in this disease. However, they also addressed that the same could happen in AD and other dementia-causing diseases, making it less useful in diagnosis, but helpful in understanding the underlying mechanisms of iNPH. Another limitation in their study is that the subjects were not well-matched in age (30).

### Dopamine Transporter Single-Photon Emission CT (DAT-SPECT)

Dopamine transporter single-photon emission enables clinicians to detect dopaminergic deficit in patients with PD and facilitate diagnosis with high sensitivity (31). It is also observed to be reduced in a great portion of patients with iNPH. In pursuit of mechanisms behind such phenomenon, Alessia et al. took the lead to conduct DAT-SPECT scans before and after surgical interventions in subjects with iNPH and indicated that such dopaminergic deficit normalized after the shunting procedure, suggesting that DAT-SPECT might be a novel approach to evaluate the progression of iNPH (32, 33).

The above modalities more or less handed scientists more clues about the pathology of NPH, including white matter ischemia, loss of elasticity in periventricular tissue, distortion in neural tracts, and impaired glymphatic clearance. They also improved our ability to distinguish NPH from other comorbidities like AD. They will continue to help with the pathology and prevention of NPH. However, the weak points and limitations in those studies should also be noted and the integrated applications of multimodal techniques and analysis on a larger scale are still in need. The pathogenesis of NPH is still far from being fully understood.

## Application of New Techniques Utilizing Artificial Intelligence

### Volume Calculation

Quon et al. achieved a deep learning model for automatic ventricle segmentation and volume calculation (34). They built a complex neural network that takes T2-weighted MRI images as input and produced satisfying accuracy in volume calculation with more rapid processing speed. This promising tool is able to facilitate the evaluation of hydrocephalus. However, the model is constructed based on the data of 200 pediatric patients, leaving uncertainty when applied to elderly patients.

### Automated Segmentation

Besides volume calculation, with the development of computational radiology, researchers can now have imaging data automatically detected or segmented for markers of interest. Gunter et al. from Mayo Clinic proved the possibility of using machine models to select DESH signs and determine ventricular volume with great accuracy (false negative ≈ 2%, false-positive ≈ 5%) (35). Similar techniques were also utilized by Peterson and his colleagues from the University of Cambridge, who found volumes of the caudate, thalamus, putamen, pallidum, and hippocampus significantly reduced in NPH, which are associated with cognitive impairment (36).

### Diagnosis Assistance

To take the topic further, Duan et al. from China reported an artificial intelligence (AI) model designed for a straightforward diagnostic purpose that uses CT images of 1,000 patients with hydrocephalus and 1,000 controls (37). The model surprisingly delivered 93.6 and 94.4% specificity with an accurate rate of 94%, higher than that of junior residents (93.4%) but still slightly lower than that of more experienced doctors. Also, this study was not specifically designed for iNPH patients.

### Outcome Prediction

Senders el at. systematically analyzed the use of machine learning (ML) methods in the field of neurosurgery, with a spotlight on surgery outcome, through literature review (38). They concluded that the ML performs spectacularly in predicting the outcome of many neurosurgery conditions, including traumatic brain injury (TBI), tumor, functional diseases, as well as hydrocephalus. Although the research did not limit the types of data used to images, it provides confidence for others that AI or ML is the future direction of neuroimaging development.

Artificial intelligence, or machine learning, a subbranch of AI, has been extensively studied in the frontiers of neuroimaging and there is a wide range of attempts that already gained gratifying results. Using non-invasive imaging data, models can be trained to handle single or mixed tasks well, providing new references for clinical practices. It surely is the next step in neuroimaging of not only hydrocephalus but also many other neurosurgery diseases.

## Summary and Future Development

Normal-pressure hydrocephalus is a relatively rare disease whose prevalence is greatly underestimated. Some patients with iNPH respond very well to shunting surgery, the first-line surgical treatment. However, selecting the to-be-benefited patients from a huge number of elderlies worldwide is challenging, highlighting the importance of better identification of suspicious patients and evaluation for prognostication. In addition to invasive measures like tap test, non-invasive imaging examination are of great value. Multiple radiological markers are summarized in this review. They were, are, and will still be the foundation of the screening process. Novel means of techniques enables us to better distinguish iNPH with its common comorbidities and even provide references for prognostication. They showed great potential in helping us understand pathophysiology. In the future, they will continue to assist with diagnostic procedures. And hopefully, using accurate imaging techniques, more detailed morphological and structural parameters will be developed. In combination with modalities that focuses on functional changes and with the help of AI, it will be easier to solve clinical uncertainty in iNPH management and distinguish it from other degenerative diseases. Unfortunately, no single or combined exams can now effectively answer all three questions—diagnosis, severity, and prognosis. Also, most novel examinations are validated in limited samples, and further comprehensive research is needed before creating a systematic approach to guide the whole course of diagnosis and treatment. Nowadays, iNPH is gaining its awareness in many centers. Hopefully this will accelerate the investigation and output of such studies.

## Author Contributions

RY and JWen wrote the manuscript. JWei edited and revised the manuscript. All authors contributed to the submission of this mini-review.

## Conflict of Interest

The authors declare that the research was conducted in the absence of any commercial or financial relationships that could be construed as a potential conflict of interest.

## Publisher's Note

All claims expressed in this article are solely those of the authors and do not necessarily represent those of their affiliated organizations, or those of the publisher, the editors and the reviewers. Any product that may be evaluated in this article, or claim that may be made by its manufacturer, is not guaranteed or endorsed by the publisher.
